# Simulation möglicher Auswirkungen des Krankenhausversorgungsverbesserungsgesetzes (KHVVG) auf die stationäre Versorgung in der HNO-Heilkunde

**DOI:** 10.1007/s00106-025-01642-z

**Published:** 2025-05-28

**Authors:** Stephan Lang, Thomas K. Hoffmann, Thomas Deitmer, Martin Jäckel, Steffen Rohwer, Stefan Mattheis, Timo Stöver, Nicole Rotter, Thomas Zahnert, Jens Peukert

**Affiliations:** 1 Klinik für Hals-Nasen-Ohren-Heilkunde, Kopf- und Hals-Chirurgie, Universitätsklinikum Essen, Universität Duisburg-Essen, Hufelandstraße 55, 45147 Essen, Deutschland; 2 Klinik für Hals‑, Nasen- und Ohrenheilkunde, Kopf- und Hals-Chirurgie, Universitätsklinik Ulm, Ulm, Deutschland; 3 Deutsche Gesellschaft für Hals-Nasen-Ohrenheilkunde, Kopf- und Hals-Chirurgie e. V., Bonn, Deutschland; 4 Klinik für Hals‑, Nasen- und Ohrenheilkunde, Kopf- und Hals-Chirurgie, Helios Kliniken Schwerin, Schwerin, Deutschland; 5 Lohfert & Lohfert AG, Hamburg, Deutschland; 6 Klinik für Hals-Nasen-Ohren-Heilkunde, Kopf- und Hals-Chirurgie, Universitätsklinikum Essen, Universität Duisburg-Essen, Essen, Deutschland; 7 Klinik für Hals‑, Nasen‑, Ohrenheilkunde, Universitätsmedizin Frankfurt, Goethe-Universität Frankfurt a.M., Frankfurt a. M, Deutschland; 8 Klinik für Hals-Nasen-Ohrenheilkunde, Kopf- und Halschirurgie, Universitätsklinikum Mannheim, Mannheim, Deutschland; 9 Klinik für Hals‑, Nasen- und Ohrenheilkunde, Kopf- und Hals-Chirurgie, Universitätsklinikum Dresden, Dresden, Deutschland

**Keywords:** Krankenhausreform, Leistungsgruppen, Strukturvoraussetzungen, Krankenhausversorgungsverbesserungsgesetz, KHVVG, Hospital reform, Medical specialties, Structural requirements, Hospital Care Improvement Act, KHVVG

## Abstract

Eine grundlegende Krankenhausreform ist u. a. aufgrund des bestehenden Fachkräftemangels, postpandemischer Produktivitätsveränderungen und einer bestehenden Erlös-Inflations-Divergenz überfällig. Ausgehend von einer in NRW angestoßenen Krankenhausreform wurde mit dem Ziel einer auskömmlicheren Finanzierung, der Sicherung und Steigerung der Behandlungsqualität sowie einer Entbürokratisierung das sog. Krankenhausversorgungsverbesserungsgesetz („KHVVG“) initiiert. Dieses ist inzwischen vom Bundesrat verabschiedet und formal ab dem 01.01.2025 in Kraft getreten. Für den Leistungsbereich HNO wurden hierbei zwei Leistungsgruppen (Allgemeine HNO, Cochleaimplantate) zur konkreten Krankenhausplanung ausgewiesen. Das Gesetz fordert ferner Mindestvoraussetzungen für stationäre Fachabteilungen (bspw. 3 HNO-Fachärzte in Vollzeit einschließlich 24/7-Rufbereitschaft) und definiert Leistungsrestriktionen bei onkochirurgischen Leistungen. Darüber hinaus werden Mindestvorhaltezahlen für alle Leistungsgruppen eingeführt. All diese Maßnahmen werden zu einer relevanten Veränderung der Krankenhauslandschaft einschließlich des Belegarztwesens und einer Zentralisierung hochspezialisierter Leistungen führen. Auch sind Effekte für die Facharztweiterbildung möglich. Zusammenfassend wird das KHVVG die stationäre Versorgung unserer Patienten deutlich verändern und erfordert daher flexible Anpassungsprozesse, denen wir uns als Fachorgane stellen müssen. Nachfolgend werden die Auswirkungen des KHVVGs für unser Fach simuliert und diskutiert, um mögliche Konsequenzen besser abschätzen und Anpassungsprozesse frühzeitig anstoßen zu können.

## Abstract (en)

A fundamental hospital reform is urgently needed due to the existing shortage of specialists, postpandemic productivity changes, and increasing financial deficits faced by hospitals in Germany. Starting from a hospital reform initiated in North-Rhine Westphalia (NRW), the so-called Hospital Care Improvement Act (“Krankenhausversorgungsverbesserungsgesetz”, KHVVG) was launched at the federal level with the aim of ensuring more adequate funding, safeguarding and improving treatment quality, and reducing bureaucracy. This was passed by the Federal Council and officially came into force on 1 January 2025. For the specialty of Otorhinolaryngology, two categories—general ENT and cochlear implants—were selected for future hospital planning. The new law also established minimum physician requirements (e.g., 3 full-time physicians including 24/7 on-call duty) and defines so-called “oncological capping” as well as minimum number of cases for each defined entity. These measures are expected to lead to significant changes in the hospital landscape, including centralization of highly specialized services, with potential implications for specialist training. In summary, the KHVVG will ultimately alter patient care, thus, requiring flexible adaptation processes, which will be supported by professional societies. For this reason, we have simulated and discussed the effects of the KHVVG on our field to better assess possible consequences and initiate adjustment processes at an early stage.

Basierend auf dem Koalitionsvertrag der Ampelkoalition aus dem Jahr 2021 wurde als erster Schritt zur Umsetzung einer Krankenhausreform vom Bundesgesundheitsminister eine Regierungskommission eingesetzt, die die Ergebnisse ihrer Arbeit als zukünftige Rahmenbedingungen für eine Gesundheitsstrukturreform vorstellte.

Ein wesentlicher Kernpunkt war dabei die Etablierung einer Leistungsgruppenstruktur, in der alle im Krankenhaus stationär behandelten Fälle einer Leistungsgruppe zugeordnet werden und die dadurch als systematische Basis einer Leistungsallokation dienen soll. Dabei werden Leistungsgruppen im Kontext des KHVVG (Gesetz zur Verbesserung der Versorgungsqualität im Krankenhaus und zur Reform der Vergütungsstrukturen – Krankenhausversorgungsverbesserungsgesetz – KHVVG; Bundesrat-Drucksache 532/24, 01.11.2024, Gesetzesbeschluss des Deutschen Bundestages) als Bündelung medizinisch ähnlicher Leistungen verstanden, für die spezifische Qualitätskriterien gelten. Die Qualitätskriterien beziehen sich im Kontext insbesondere auf Struktur- & Prozessqualität. Darüber hinaus wurde eine Finanzierungsreform vorgeschlagen, die das reine Fallpauschalensystem ablösen soll. Teile der Vergütung sollen künftig als sogenannte Vorhaltekosten – gekoppelt an einen vergebenen Leistungsauftrag – fallunabhängig ausgezahlt werden [1].

Das Krankenhausversorgungsverbesserungsgesetz (KHVVG) passierte nach zwei Jahren intensiver Diskussion zwischen Bund und Ländern am 22.11.2024 den Bundesrat. Während der nächsten Jahre werden die Finanzierung der Krankenhäuser schrittweise umgestellt und eine Spezialisierung auf bestimmte Leistungsgruppen eingeführt, flankiert durch Qualitätsvorgaben wie Mindestzahlen und Personaluntergrenzen. Des Weiteren wird im novellierten § 40 KHG ein Mechanismus zum onkochirurgischen Capping vorgestellt. Dieser wird auch auf das Fachgebiet der HNO-Heilkunde Implikationen haben. Die „kleinsten“ Versorger, die gemeinsam weniger als 15 % der noch zu definierenden onkochirurgischen Leistungen im Fachgebiet erbringen, sollen zukünftig diese Fälle nicht mehr versorgen und werden nicht mehr für die Leistungserbringung vergütet.

Der nachfolgende Artikel simuliert potenzielle Auswirkungen des KHVVG auf die stationäre HNO-Heilkunde im Hinblick auf Krankenhauszahlen, Vorhaltung von Personal und Strukturen sowie auf Weiterbildungsoptionen.

## Methode

Die Regelungen des KHVVG (Qualitätsvorgaben) wurden auf eine bundesweite Datenbasis aus multiplen Datenquellen angewendet. Zur Berechnung der Fallvolumina wurde auf den Leistungsgruppen-Algorithmus aus NRW zurückgegriffen, da der InEK-Leistungsgruppen-Grouper zum Zeitpunkt der Erstellung dieser Arbeit noch nicht vorlag. Die Datenquellen sind einerseits die Qualitätsberichte der Krankenhäuser und die darin veröffentlichten Leistungs- und Strukturangaben aus dem Jahr 2023. Andererseits wurden die bundesweiten Leistungsdaten, die sogenannte DRG-Statistik aus dem Jahr 2023, veröffentlicht vom Statistischen Bundesamt, herangezogen. Darüber hinaus wurde für spezifische Analysen auf den anonymisierten Lohfert & Lohfert Klinikvergleich, der auf §21-Abrechnungsdaten basiert, zurückgegriffen.

Als Grundlage für die Simulationen wurden einige retrospektive Analysen durchgeführt, die die Leistungsentwicklung der stationären HNO-Heilkunde in Deutschland insbesondere in den Jahren 2019 bis 2023 zeigen.

## Bundesweite stationäre Leistungsentwicklung

In allen deutschen Bundesländern war ein pandemiebedingter Fallzahlrückgang über das gesamte medizinische Leistungsspektrum festzustellen. Im Vergleich zu 2019 gab es in Deutschland einen nachhaltigen Einbruch der Fallzahlen um 11,66 % über alle Bundesländer und stationäre Leistungen hinweg. Im Vergleich zu 2022 stiegen die Fallzahlen 2023 insbesondere im Nordwesten Deutschlands wieder an. Die Fallzahl in der für die Hals-Nasen-Ohren-Heilkunde bedeutendsten MDC (Major Diagnostic Category) 03 „Krankheiten und Störungen des Ohres, der Nase, des Mundes und des Halses“ hatte in den Jahren vor der Pandemie eine leichte Wachstumsentwicklung (2010 bis 2016: +4 %) gezeigt, die aber unter dem mittleren Marktwachstum lag. Der Einbruch in der Pandemie von 2019 auf 2020 ist mit 22 % besonders stark ausgefallen. Seit 2020 zeigt sich eine leichte Erholung der Fallzahlen. Die MDC 03 liegt mit −13 % aber weiter unter dem präpandemischen Niveau und ist stärker betroffen als andere medizinische Fachgebiete (durchschnittlich −11,66 %). Bei Betrachtung des Casemix zeigte sich zwischen 2010 und 2016 ein Wachstum von 6,4 %, welches 2,4 Prozentpunkte höher ausfiel als das der Fallzahlen.

**Abb. 1:**
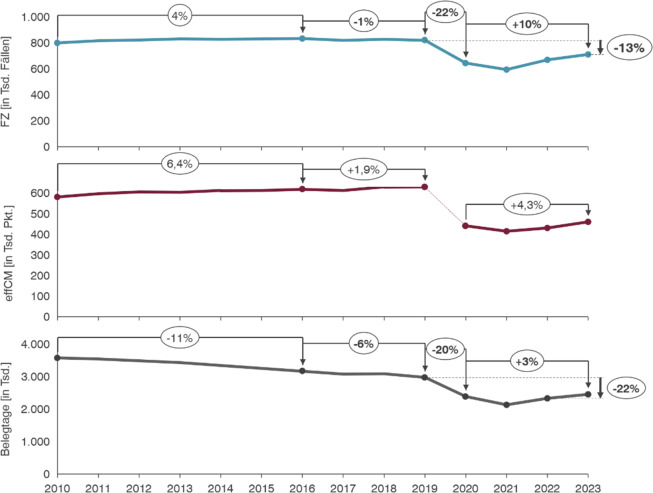
Entwicklung Fallzahl, effCM, Belegtage im MDC 03 (2010–2023)

**Abb. 2:**
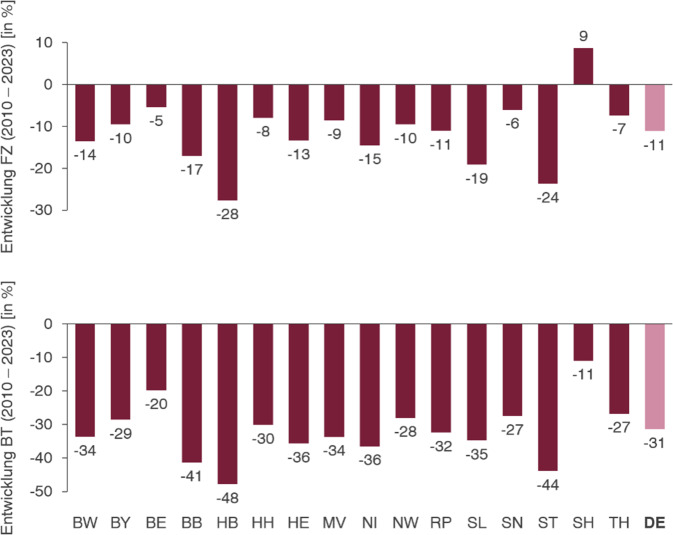
Relative Entwicklungen MDC 03 nach Bundesland (2010–2023)

Auch in Bezug auf den Casemix hatte die Pandemie gravierende Auswirkungen. Diese können aufgrund der Anpassung der DRG-Systematik durch die Ausgliederung des Pflegebudgets nicht quantifiziert werden. Nach der Pandemie zeigte sich eine moderate Erholung des Casemix (2020 bis 2023: +4,3 %). Eine besonders eindrückliche Entwicklung fand sich für die Hals-Nasen-Ohren-Heilkunde bei Betrachtung der Belegungstage. Im Vergleich zu 2010 haben sich diese im Jahr 2023 um 35 % reduziert. Zwischen 2010 und 2016 ist trotz relevanten Fallzahl-Wachstums ein deutlicher Rückgang an Belegungstagen festzustellen. Diese Beobachtung korreliert mit einem signifikanten Rückgang der Verweildauer von ca. 15 %. Pandemiebedingt zeigt sich mit 22 % ein stärkerer Rückgang der Belegungstage als nach Fallzahl (−13 %). Zuletzt nimmt die Belegung in der betrachteten MDC 03 wieder leicht zu. In Summe ist ein Belegungstage-Rückgang von 35 % im Zeitraum 2010 bis 2023 festzustellen, der zum überwiegenden Teil auf die Zeit seit 2019 zurückzuführen ist (−22 %). Die Entwicklungen sind in Abb. 1 dargestellt. In den Entwicklungen der einzelnen Bundesländer sind sowohl hinsichtlich der Fallzahl als auch der Belegungstage relevante Unterschiede erkennbar. Bei der Fallzahl zeigte sich ein durchschnittlicher Rückgang von 11 %. Die Spannbreite der Entwicklung in den einzelnen Ländern ist dabei groß. Während die Fallzahl in Schleswig-Holstein um 9 % gewachsen ist, fällt diese in Bremen mit −28 % am stärksten ab. Nach Belegungstagen zeigte sich in allen Bundesländern ein negativer Trend. Der Rückgang der Belegungstage fiel mit −11 % in Schleswig-Holstein am geringsten und in Bremen mit −48 % am deutlichsten aus. Auch andere Länder wie Brandenburg (−41 %) und Sachsen-Anhalt (−44 %) zeigten einen gravierenden Rückgang der Belegung im beobachten Zeitraum. Die Entwicklungen nach Fallzahl & Belegtagen für alle Bundesländer sind in Abb. 2 dargestellt [2].

## KHVVG-Leistungsgruppen im Bereich HNO

Das KHVVG sieht für die Hals-Nasen-Ohren-Heilkunde zwei Leistungsgruppen vor. Die *allgemeine Leistungsgruppe „HNO“* (50 bzw. 24.1 lt. NRW-Krankenhausplan), die über den Fachabteilungsschlüssel definiert ist, bildet den überwiegenden Anteil der Fälle im Fachgebiet ab. Die *Cochleaimplantate* werden einer separaten Leistungsgruppe zugeordnet (51 bzw. 24.2 lt. NRW-Krankenhausplan). Eine exakte Definition gemäß KHVVG liegt zum Erstellungszeitpunkt nicht vor, es wird jedoch angenommen, dass die Definition der Leistungsgruppen keine diametralen Unterschiede zeigen wird. Die NRW-Definition der Leistungsgruppen ist in Tab. 1 abgebildet.

**Tab. 1:** Leistungsgruppendefinition gemäß NRW-Krankenhausplan (Stand: Oktober 2024)

LG-Nr.	LG-Name	Definition der Leistungsgruppe
50 bzw. 24.1	HNO	*Allgemeine Leistungsgruppe (definiert gemäß FAB-Schlüssel):* FAB 2600 FAB 2690
51 bzw. 24.2	Cochleaimplantate	*Spezifische Leistungsgruppe (definiert gemäß OPS-Code):* 5‑209.2 5‑209.7 5‑209.8

Zur Beurteilung des Leistungsgruppenspektrums einer HNO-Abteilung wurde auf den Lohfert & Lohfert Klinikvergleich zurückgegriffen, da diese anonymisierte Datenquelle (basierend auf §21-Datensätzen) fallbezogene Analysen ermöglicht. Für die Beantwortung der Fragestellung wurde eine Stichprobe nach spezifischen Kriterien selektiert, die einen Umfang von ca. 385.000 Fällen in über 100 Krankenhäusern aller Versorgungsstufen umfasst. Die Leistungsgruppen HNO & Cochleaimplantate machen an den durch eine HNO-Fachabteilung entlassenen Fälle einen Anteil von 99,79 % aus. Daraus folgt, dass die im Rahmen des NRW-Krankenhausplans festgelegte Leistungsgruppen-Hierarchie keine relevanten Fallvolumina aus der Hals-Nasen-Ohren-Heilkunde betrifft. Nahezu alle HNO-assoziierten Fälle werden auch einer der beiden definierten Leistungsgruppen zugeordnet. Die möglichen Auswirkungen des neu entwickelten Leistungsgruppen-Groupers gemäß KHVVG wurden hier nicht berücksichtigt. Neben der HNO und den Cochleaimplantaten tauchen regelhaft (aber sehr selten) einzelne Fälle der LG „Perinatalzentrum Level 2“ auf. Singulär (Fallvolumen < 0,1 %) werden auch Fälle anderer Leistungsgruppen über die HNO entlassen. Aufgrund der sehr geringen Fallzahlen werden diese Effekte als nachrangig betrachtet. Im Fachgebiet HNO-Heilkunde ergibt sich für die spezifische Leistungsgruppe Cochleaimplantate, dass nahezu 98 % der Fälle über die Fachabteilung HNO entlassen werden. Die Analyse zeigt partiell Entlassungen von Fällen, die in die Leistungsgruppe Cochleaimplantate fallen und über pädiatrische Fachabteilungen entlassen werden. Das Gesamtvolumen dieser Fälle ist mit unter 2,5 % nachrangig zu bewerten.

Die im Rahmen des KHVVG festgelegten Qualitätskriterien für die Leistungsgruppen sind in Tab. 2 dargestellt. Dabei gibt es Mindestkriterien, ohne die keine Zuweisung der Leistungsgruppe erfolgen kann, und Auswahlkriterien, die bei Auswahlentscheidungen der Planungsbehörden anzuwenden sind. Die im Rahmen des KHVVG neu hinzugekommene begrenzte Anrechenbarkeit einzelner Ärzte auf maximal drei Leistungsgruppen hat für das Fachgebiet HNO-Heilkunde keine Implikationen, da lediglich die beiden Leistungsgruppen der Hals-Nasen-Ohren-Heilkunde den entsprechenden Facharzttitel in ihren Definitionen erfordern. Die Leistungsgruppen sind über Mindest- und Auswahlkriterien untereinander vernetzt (Tab. 2). So benötigt die LG HNO unter anderem die Leistungsgruppen Allgemeine Innere Medizin und Allgemeine Chirurgie. Umgekehrt kann beispielsweise die LG Stammzelltransplantation nicht ohne die LG HNO (wenigstens in Kooperation) erbracht werden. Die in Tab. 2 dargestellten Auswahlkriterien sind nur bei einer Auswahlentscheidung der Landesbehörde anzuwenden.

**Tab. 2:** Qualitätskriterien Leistungsgruppen gemäß KHVVG-Strukturen der stationären HNO-Heilkunde

LG-Nr.	LG-Name	Erbringung verwandter LG	Vorhaltung relevanter Geräte	Fachärztliche Vorgaben
50 bzw. 24.1	HNO	*Mindestkriterium:* LG Allg. Chirurgie LG Allg. Innere Med. LG Intensivmedizin	*Mindestkriterium:* Elektrische Reaktionsaudiometrie	*Mindestkriterium:* Mindestens 3 VZÄ FA Hals-Nasen-Ohren-Heilkunde Rufbereitschaft jederzeit
		*Auswahlkriterium:* LG Allg. Kinder- & Jugendmedizin LG MKG (in Kooperation)	*Auswahlkriterium:* MRT PET-CT Doppler- & Duplexsonographie	*Auswahlkriterium:* ZW-Allergologie
51 bzw. 24.2	Cochleaimplantate	*Mindestkriterium:* LG Allg. Chirurgie LG Allg. Innere Med. LG Intensivmedizin LG HNO	*Mindestkriterium:* Elektrische Reaktionsaudiometrie	*Mindestkriterium:* Mindestens 3 VZÄ Hals-Nasen-Ohren-Heilkunde Rufbereitschaft jederzeit
		*Auswahlkriterium:* LG Allg. Kinder- & Jugendmedizin LG MKG (in Kooperation)	*Auswahlkriterium:* MRT PET-CT Doppler- & Duplexsonographie	*Auswahlkriterium:* FA Phoniatrie & Pädaudiologie

**Abb. 3:**
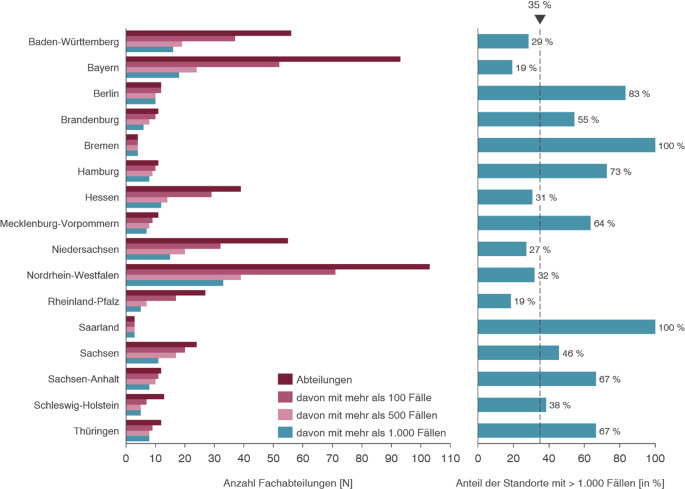
Versorgungsstrukturen nach Bundesland in der HNO. Hinweis: Die Darstellung der absoluten Anzahl der HNO-Abteilungen mit mehr als 100/500/1000 Fällen in Abb. 3 erfolgt dabei stets als Teilmenge der Grundgesamtheit. Daraus folgt, dass beispielsweise die Versorger mit > 1000 Fällen auch in der Menge der Versorger mit > 500 Fällen enthalten sind

Die Abb. 3 zeigt eine Analyse der bestehenden Fachabteilungsstruktur im Fachgebiet HNO-Heilkunde. Dabei wird die Zahl der Abteilungen in Fallzahlclustern (mehr als 100, 500 oder 1000 Fällen pro Jahr) je Bundesland dargestellt.

Die HNO-Fachabteilungen weisen im Durchschnitt ca. 974 Fälle je Standort an 486 Standorten auf. Die Analyse zeigt signifikante Unterschiede in der Größe der Fachabteilungen. Ein überproportional großer Anteil kleiner Abteilungen korreliert dabei mit einer erhöhten Dichte (kleiner) belegärztlicher Abteilungen im jeweiligen Bundesland.

Betrachtet man den Anteil der Versorger mit einer Fallzahl über 1000 zeigt sich, dass dieser ebenfalls regional auf Bundeslandebene erheblich schwankt. Im Durchschnitt versorgen dabei 35 % der Fachabteilungen über 1000 Fälle im Jahr 2023. In Bremen und im Saarland liegt der Anteil der Versorger über 1000 Fälle beispielsweise bei 100 %, in Rheinland-Pfalz & Bayern dagegen nur bei 19 %.

Diese Resultate belegen, dass in der Vergangenheit bundeslandspezifisch stark abweichende Leistungskonzentrationen im Rahmen der Krankenhausplanung für das Fachgebiet erfolgt sind. Die unterschiedliche Versorgungsstruktur und der unterschiedliche regionale Anteil von Versorgern mit über 1000 Fällen wird in Abb. 3 dargestellt.

## Prüfung der fachärztlichen Ausstattung gemäß KHVVG

Die Qualitätskriterien für die Leistungsgruppe HNO wurden bereits zuvor erläutert. Für das Fachgebiet der Hals-Nasen-Ohren-Heilkunde ist insbesondere die fachärztliche Ausstattung von Relevanz. Beide HNO-Leistungsgruppen sehen eine minimale fachärztliche Ausstattung von drei Vollzeitäquivalenten „FA Hals-Nasen-Ohren-Heilkunde“ vor. Sofern für die entsprechende Leistungsgruppe eine belegärztliche Leistungserbringung vorgesehen ist, wird eine Belegärzteschaft aus Ärzten benötigt, die drei Vollzeitäquivalente abbilden. Dabei ist die Erfüllung der personellen Anforderungen entweder durch Belegärzte oder durch am Krankenhaus angestellte Ärzte vorgesehen. Um die Auswirkungen der Qualitätskriterien auf das Fachgebiet zu analysieren, wurde auf die Angaben aus den Qualitätsberichten (2023) zurückgegriffen.

**Abb. 4:**
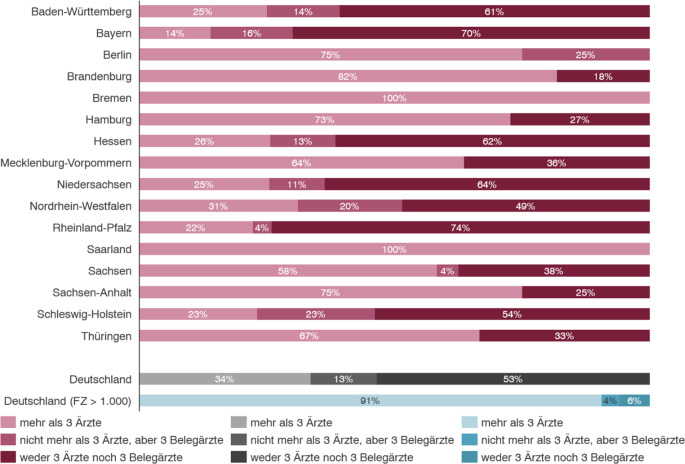
Prüfung der KHVVG-gemäßen fachärztlichen Anforderungen HNO

Im Status quo weisen von den 486 Standorten, die die Fachabteilung HNO vorhalten, 260 und damit ca. 53 % der Leistungserbringer weder die erforderlichen drei Fachärzte noch drei Belegärzte auf. Mit angestellten Fachärzten werden die Qualitätsvorgaben der personellen Anforderung sogar nur von 163 Standorten bzw. 34 % erfüllt. In Abb. 4 werden alle Fachabteilungen in Deutschland basierend auf der Erfüllung der Qualitätsvorgaben der Leistungsgruppe dargestellt. Im Vergleich aller Standorte mit den Standorten, die über 1000 Fälle pro Jahr haben, zeigen sich deutliche Unterschiede. Bei den Versorgern mit einem Leistungsvolumen über 1000 Fällen erfüllen 159 der 169 Standorte und somit 94 % die personellen Anforderungen. Lediglich sechs (unter 5 %) der Standorte erfüllen hier die Qualitätskriterien laut dem Qualitätsbericht nicht. Diese Ausreißer können auf Datenqualität der Qualitätsberichte zurückführbar sein. Für große Fachabteilungen ist die Qualitätsvorgabe somit in der Regel unproblematisch erfüllbar.

Auch bei dieser Analyse weisen die einzelnen Bundesländer diametrale Unterschiede auf. Bei Betrachtung der Grundgesamtheit der HNO-Fachabteilungen im regionalen Vergleich zeigen sich Zusammenhänge zwischen dem Anteil der großen Fachabteilungen mit > 1000 Fällen je Bundesland und den Erfüllungsgraden der Personalanforderung der Leistungsgruppe. Daraus resultiert die Hypothese, dass eine flächendeckende Erfüllung der Qualitätsvorgaben in einer Struktur mit vielen kleinen Fachabteilungen schwer umzusetzen ist. Bundesländer, in denen die Leistungen der HNO-Heilkunde bereits gut zentralisiert wurden, wie bspw. Bremen, Berlin, Saarland, Sachsen-Anhalt und Hamburg, haben im länderspezifischen Bereich weniger Probleme bei der Erfüllung der Qualitätsvorgaben gemäß KHVVG. Die Analyse der Daten in Abb. 4 zeigt, dass in Bremen, Berlin und im Saarland bereits heute alle Versorger die fachärztlichen Vorgaben erfüllen. In den Bundesländern Brandenburg, Sachsen-Anhalt sowie Hamburg werden diese Vorgaben derzeit schon von über 70 % der derzeitigen Leistungserbringer erfüllt.

In Rheinland-Pfalz, Niedersachsen, Baden-Württemberg und Bayern zeigen über 60 % der Standorte derzeit nicht erfüllte Qualitätsvorgaben. Insgesamt erfüllen in zehn Bundesländern über 30 % der derzeitigen Versorger nicht die im Rahmen des KHVVG angestrebten Qualitätsvorgaben.

Diese heterogene Versorgungsstruktur impliziert bundeslandspezifische Konsequenzen. Gravierende Auswirkungen für die Weiterbildung werden sich absehbar nicht ergeben, da die großen stationären Versorger in der Vergangenheit eine dominante Rolle in der Weiterbildung gespielt haben. Somit dürfte der Wegfall von Belegabteilungen oder kleinen stationären Einrichtungen nur einen geringen Einfluss auf die Anzahl der Weiterbildungsstellen haben. Andere Mechanismen, wie das onkochirurgische Capping, könnten die komplette Weiterbildung, aufgrund eines unvollständigen komplexchirurgischen Leistungsspektrums dieser Weiterbildungseinrichtung, erschweren. Der Berechnungsmechanismus und potenzielle Auswirkungen werden in einem folgenden Kapitel separat analysiert.

## Mindestvorhaltezahlen für die HNO-Leistungsgruppen

Das KHVVG sieht eine noch zu definierende Mindestfallzahl stationärer Behandlungen in allen Leistungsgruppen vor. Diese werden in § 135f SGB V als „Mindestvorhaltefallzahlen“ (MVHZ) vorgestellt. Eine genaue Vorhersage zukünftiger Mindestvorhaltezahlen ist derzeit noch nicht möglich, da das KHVVG hierzu keine exakten Berechnungsvorgaben macht, sondern lediglich den Berechnungsmechanismus vorstellt. Weitere Konkretisierungen über Perzentile je Leistungsgruppe sind noch festzulegen. Die hier vorgestellte Kalkulation beruht auf den Daten im Qualitätsbericht 2023, aus denen für die Leistungsgruppen des Fachgebiets für alle Standorte Fallzahlen abgeleitet werden können.

Dabei wird auf das in der Gesetzesbegründung des KHVVG beispielhaft genannte 20. Perzentil aller in der Leistungsgruppe versorgten Fälle zurückgegriffen: Zur Bestimmung der Mindestvorhaltezahl basierend auf dem festgelegten Perzentil wird die kumulierte Fallzahl der „kleinsten“ Versorger herangezogen, bei der das Perzentil der Gesamtfallzahl erreicht wird. Potenziell wären für die Leistungsgruppen im Fachgebiet der HNO-Heilkunde auch andere Perzentile denkbar.

**Abb. 5:**
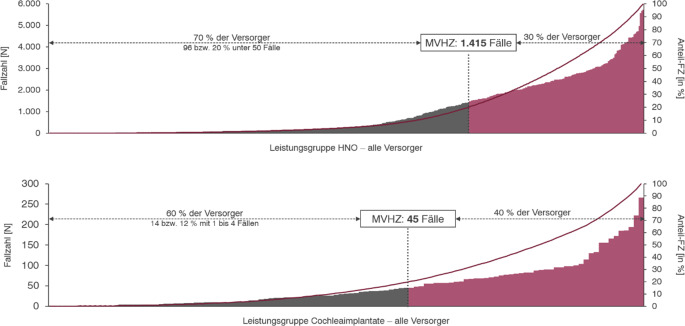
Mindestvorhaltezahlen nach KHVVG in der HNO

Aus dieser Analyse resultiert, wie in Abb. 5 dargestellt, für die Leistungsgruppe HNO-Heilkunde eine Mindestvorhaltezahl von 1415 Fällen (20. Perzentil). Der Spitzenversorger versorgt dabei über 5700 Fälle. Bei Anwendung des 20. Perzentils würden 70 % der Abteilungen, die gemeinsam 20 % des Patientenvolumens versorgen, unter der MVHZ liegen. Von diesen Versorgern haben 96 bzw. 20 % der Grundgesamtheit ein Leistungsvolumen unter 50 Fällen pro Jahr. Wenn der Gesetzgeber sich für ein anderes Perzentil entscheidet, dann kann diese Fallzahl abweichen. Für das 10. Perzentil ergibt sich beispielsweise eine Vorhaltefallzahl von ca. 800 Fällen, während eine noch drastischere Festlegung auf das 30. Perzentil sogar 1850 Fälle als Mindestfallzahl ergeben würde.

In der Leistungsgruppe Cochleaimplantate ergibt sich basierend auf dem 20. Perzentil eine Mindestvorhaltezahl von 45 Fällen. Der Spitzenversorger versorgt in dieser Leistungsgruppe mit 266 Fällen nahezu 5 % des deutschlandweiten Fallvolumens. Eine Mindestfallzahl in dieser Höhe würde die Versorgung auf die derzeit 40 % größten Versorger konzentrieren. Sollte ein anderes Perzentil gewählt werden, kann dies einen starken Einfluss auf die Höhe der MVHZ haben. Für das 10. Perzentil ergibt sich beispielsweise eine Vorhaltefallzahl von ca. 26 Fällen, während eine noch drastischere Festlegung auf das 30. Perzentil sogar in 66 Fällen als MVHZ resultieren würde.

Daraus resultieren signifikante Veränderungen der Versorgungslandschaft. Die gesamte Patientenwanderung beträgt bei einem 20. Perzentil als MVHZ ca. 20 % der Fälle in der Leistungsgruppe. Für die Leistungsgruppe HNO würde dies ca. 92.000 Fälle und für die Gruppe Cochleaimplantate ca. 1200 Fälle ergeben, die von den verbleibenden Versorgern mit Zuweisung der Leistungsgruppe mitversorgt werden müssten. Ungefähr 70 % der derzeitigen Versorger in der stationären Hals-Nasen-Ohren-Heilkunde würden unter Berücksichtigung der o. a. Annahmen keinen Versorgungsauftrag im Rahmen der Leistungsgruppe bekommen und folglich von der Leistungserbringung ausgeschlossen werden.

## Beschränkungen onkochirurgischer Leistungen

Die im Kontext des KHVVG genannte onkologische Leistung ist bislang nicht abschließend definiert und wird durch das Bundesinstitut für Arzneimittel und Medizinprodukte (BfArM) zu einem späteren Zeitpunkt festgelegt. Das BfArM erstellt eine Liste mit relevanten ICD- und OPS-Kombinationen, auf deren Grundlage das InEK die entsprechenden Fallzahlen für die definierten onkochirurgischen Leistungen ermittelt. Die Ermittlung der Fallzahl basiert, gemäß dem im KHVVG beschriebenen Algorithmus, auf der Fallzahl des kleinsten Versorgers, mit dem kumulativ 15 % der Fälle in dem definierten Leistungssegment erreicht werden.

Da die Qualitätsberichte keinen Fallbezug bieten, ist eine Berechnung von Fallzahlen mit ICD/OPS-Bezug nicht möglich. Für eine indikative Analyse wurden drei OPS-Codes beispielhaft als Abbild der Fallzahl ausgewählt. Bei der Auswahl der OPS-Codes wurde darauf geachtet, dass diese nahezu ausschließlich bei Tumordiagnosen zur Anwendung kommen. Eine Auswertung auf ICD-Ebene ist in der Hals-Nasen-Ohren-Heilkunde weniger zielführend, da hier sowohl operative als auch konservative Behandlungsformen zum Einsatz kommen. Eine reine Betrachtung der ICD-Kodierung stellt demnach nicht hinreichend verlässlich die Auswirkungen der 85%-Regelung in der Onkochirurgie dar. Insgesamt ist davon auszugehen, dass die Definition onkochirurgischer Leistungen abschließend nicht auf Ebene einzelner OPS/ICD-Kombinationen erfolgen wird, sondern sich an einer größeren Sammlung von HNO-spezifischen onkochirurgischen Fallgruppen (z. B. Definition Kopf-Hals-Tumorzentrum nach Onkozert) orientieren wird.

Die vorliegenden Analysen dienen dazu, die potenziellen Auswirkungen des im Gesetz vorgeschlagenen Algorithmus für die Onkochirurgie auf spezifische Fallgruppen und damit auf die Versorgungslandschaft in der HNO-Heilkunde zu beurteilen. Im Mittelpunkt stehen dabei die OPS-Codes:

- 5‑295 (Partielle Resektion des Pharynx – Pharynxteilresektion)
- 5‑403 (Radikale zervikale Lymphadenektomie – Neck-Dissection mit über drei Regionen)
- 5‑303 (Laryngektomie)

Die Anwendung der geplanten Regelung mit dem OPS-Code 5‑295 (Partielle Resektion des Pharynx – Pharynxteilresektion) zeigt, dass etwa 50 % der Einrichtungen rund 85 % aller kodierten Fälle (bzw. Prozeduren) und die übrigen 50 % der Versorger nur 15 % der Fälle versorgen. Letzteres entspricht ca. 80 Krankenhäusern. Diese Verteilung mündet in eine kritische Fallzahl von acht Eingriffen pro Haus: Jene Krankenhäuser, die mehr als acht kodierte Fälle mit dem OPS 5‑295 aufweisen, gehören zu der Hälfte der Einrichtungen, die insgesamt 85 % aller Fälle abdecken und gemäß der im KHVVG geplanten Regelung auch zukünftig zur Abrechnung der entsprechenden Leistungen ermächtigt sind.

Für den OPS 5‑403 (Neck-Dissection) ergeben sich noch gravierendere Auswirkungen. Bei diesem OPS ist eine Einschränkung der Subcodes des OPS sinnvoll. Da ein Fallbezug im Qualitätsbericht nicht möglich ist, sollen nur die OPS-Codes ausgewertet werden, die nicht regelhaft in Zusammenhang mit nichtonkologischen Diagnosen kodiert werden. Wertet man den OPS-Code 5‑403 ohne die Endsteller „.00“, „.01“, da diese auch regelhaft in Fällen ohne onkologische Diagnose angewendet werden, aus, so ergibt sich ein Mindestvolumen von 34 Fällen/Prozeduren. Diese Leistungen werden verstärkt von großen Versorgern erbracht, sodass lediglich 36 % der Versorger gemeinsam 85 % des Gesamtvolumens versorgen.

**Abb. 6:**
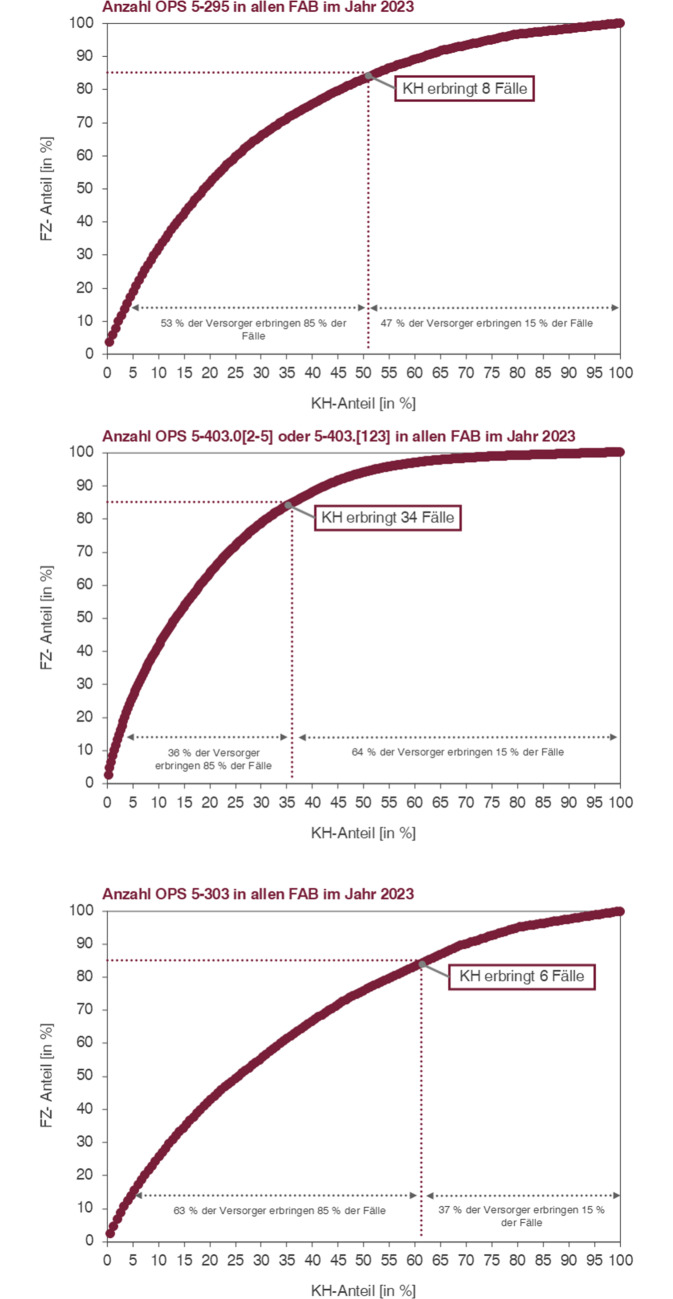
Darstellung der Verteilung ausgewählter onkochirurgischer OPS-Codes (deutschlandweit)/*FAB =* Fachabteilungen*. *Angegebene Fallzahl ist die Grenzfallzahl zur Leistungserbringung

Bei der Laryngektomie (OPS-Code 5‑303) zeigt sich ein deutlich flacherer Verlauf der kumulativen Leistungserbringung. Für diese Leistung liegt die kritische Fallzahl bei 6 Fällen, wobei ungefähr 63 % der Versorger zusammen 85 % der Prozeduren durchführen. Knapp über 50 Krankenhäuser erbringen bei dieser Prozedur gemeinsam nur 15 % der Fälle und wären zukünftig nicht mehr zur Abrechnung berechtigt. In Abb. 6 werden die kumulativen Fallzahlkurven der drei gewählten Analysen dargestellt.

## Diskussion

Bereits vor der Corona-Pandemie (bis einschließlich 2019), aber insbesondere ab 2020 waren die stationären Fallzahlen und auch die damit verbundenen Belegungstage von Patienten deutlich rückläufig. Der Bettenkapazitätsbedarf der HNO-Abteilungen sank damit deutlich. Parallel gingen auch die Fallzahlen der HNO-Abteilungen zurück [3]. Durch diese Veränderungen und durch die Vielzahl sehr fallzahlschwacher Belegabteilungen reduzierte sich die durchschnittliche Größe der Abteilungen auf ein Maß, das eine wirtschaftliche Leistungserbringung insbesondere bei den Hauptabteilungen nur schwer möglich bzw. unmöglich macht. Einschränkend muss man für die Interpretation der Zahlen anfügen, dass die bisherigen Statistiken 2023 enden. Da die Pandemie bis 2022 andauerte, kann nicht genau bestimmt werden, ob der Erholungseffekt 2023 bereits abgeschlossen war, weiter anhält oder ausbleibt.

Das KHVVG sieht für die HNO aktuell 2 Leistungsgruppen vor. Mit diesen beiden Leistungsgruppen werden das Leistungsspektrum und auch der Leistungsumfang aktueller HNO-Abteilungen sehr realitätsnah beschrieben. Alle Fälle, die derzeit in der HNO versorgt werden, fallen in eine der beiden Leistungsgruppen. Schnittstellen finden sich insbesondere zu Abteilungen für Mund‑, Kiefer- und Gesichtschirurgie. Durch die Systematik der Zuordnung in allgemeinen Leistungsgruppen wird die jeweils entlassende Fachabteilung der entsprechenden Fallpauschalen abgebildet.

Die mit der Leistungserbringung in einer MKG-Abteilung verbundene KHVVG-Leistungsgruppe MKG (mindestens Kooperationsvertrag) ist ein Auswahlkriterium als verwandte Leistungsgruppe (LG) für die Zuweisung der LG HNO. Ein weiteres Auswahlkriterium im Bereich der verwandten Leistungsgruppen ist die Leistungsgruppe Kinder- & Jugendmedizin (am Standort). Insgesamt ist davon auszugehen, dass die Auswahlkriterien für die LG HNO im ersten Schritt in den meisten Bundesländern keine gravierenden Auswirkungen haben werden.

In Zukunft sollten eine weitere Spezifizierung der Leistungsgruppen angestrebt und die Spezifikation der bestehenden Leistungsgruppen z. B. in Hinblick auf Qualitätsvorgaben optimiert werden. Dies kann im ersten Schritt auch mit dem aktuell in Erarbeitung befindlichen Zuordnungsalgorithmus („Leistungsgruppen-Grouper“), der die Zuordnung einzelner Fälle zu einer spezifischen Leistungsgruppe abbildet, geschehen. Es gibt Übergangsfristen, und die „Scharfschaltung“ des Leistungsgruppensystems wird absehbar 2027 erfolgen, wenn nicht die Länder den Prozess beschleunigen wollen.

Die absehbarste Qualitätsanforderung wird sicher die fachärztliche Ausstattung der Abteilungen werden. Dies trifft sowohl die Hauptabteilungen mit den dann nötigen 3 vollzeitäquivalenten Fachärzten als auch Belegabteilungen mit den dann notwendigen 3 involvierten Fachärzten (VZÄ) als Belegärzte. In der hier durchgeführten Simulation wird durch diese Qualitätsvorgabe ein relevanter Anteil der aktuellen HNO-Abteilungen inkl. Belegabteilungen im Zuge der neuen Krankenhausplanungen voraussichtlich nicht mehr an der Versorgung teilnehmen können.

Für die CI-Versorgung sollte eine Überarbeitung der Qualitätskriterien entsprechend der geltenden Leitlinie mit Übernahme der Mindestanforderungen erfolgen.

Mit den simulierten Veränderungen und der daraus ableitbaren deutlichen Reduktion stationärer HNO-Abteilungen mit vollem Versorgungsspektrum (inkl. Onkochirurgie) könnten auch Veränderungen für die Facharztweiterbildung resultieren. Die Befugnis zur HNO-Facharztweiterbildung wird absehbar den behandelten Fällen einer Einrichtung folgen: Entsprechend dem Portfolio einer Abteilung könnten hier kooperative Weiterbildungsformen an Bedeutung gewinnen.

Durch die beschriebenen Veränderungen wird die Bedeutung von Universitätskliniken und großen Maximalversorgern noch wichtiger werden. Fraglich ist dabei, ob für die Zentralisierung von Leistungen an den großen Kliniken genügend Ressourcen für die Behandlung der Fälle zur Verfügung stehen bzw. kurzfristig zur Verfügung gestellt werden können. Denkbar ist aber auch eine Entwicklung von HNO-Abteilungen ohne onkologische Chirurgie, die dafür hohe Fallzahlen in der HNO-Grundversorgung erbringen. Hierbei besteht allerdings das Risiko einer Spaltung des Fachgebiets und der unwirtschaftlichen Betriebsführung einer derart veränderten HNO-Klinik. Ergänzende Lösungsansätze könnten auch im Ausbau von Kooperationsmodellen liegen.

Durch die noch sehr undifferenziert vorliegenden Regelungen für onkochirurgische Leistungen kann es zu massiven Veränderungen der Behandlung von malignen Erkrankungen in der HNO-Heilkunde kommen. Die Auswirkungen werden sehr wesentlich von der Definition der noch offenen Durchführungsbestimmungen zum KHVVG abhängen.

Das Ziel der Entökonomisierung wird durch das KHVVG absehbar nicht erreicht werden. Die Finanzierungsvorgaben sind komplex und letztendlich doch an Fallzahlen gebunden. Zudem ist keine Erhöhung des Krankenhaus-Gesamtbudgets vorgesehen, weshalb lediglich eine Umverteilung der Ressourcen stattfinden wird.

Eine Entbürokratisierung ist durch das KHVVG ebenfalls nicht zu erwarten. Vielmehr zeichnet sich eine deutliche Zunahme notwendiger Dokumentationspflichten ab.

Das Ziel der Sicherung und Steigerung der Behandlungsqualität findet seinen Einfluss in der Definition von Leistungsgruppen für die Krankenhausplanung u. a. durch Mindestvorhaltezahlen und der onkochirurgischen Leistungsbeschränkung mit konsekutiver Zentralisierung.

Ausblick: Eine Evaluation der Leistungssituation wird alle zwei bis drei Jahre erfolgen. Dabei werden Änderungen der Fallzahlen von mehr als ± 20 % in die Kalkulation der Vorhaltevergütung eingehen. Vermutlich wird es in der Zukunft weitere HNO-Leistungsgruppen bspw. in der Onkologie geben.

## Fazit

Das KHVVG sieht für die HNO derzeit zwei Leistungsgruppen vor. Die Qualitätsanforderungen für die „Allgemeine HNO“ definieren sich primär über die Zahl von 3,0 Fachärzten (Vollzeitäquivalente) bei Hauptabteilungen sowie ebenfalls drei Vollzeitäquivalenten bei Belegabteilungen. Die aktuelle Versorgungssituation ist inhomogen mit regional sehr unterschiedlich ausgebildeten belegärztlichen Strukturen, die durch die Reform deutlich reduziert werden dürften. Abteilungen mit über 1000 Fällen dürften nicht betroffen sein. Die Versorgungsrolle der Universitätskliniken wird noch wichtiger werden, negative Auswirkungen auf Forschung und Lehre sind nicht zu erwarten.

## Data Availability

Die in dieser Studie erhobenen Datensätze können auf begründete Anfrage beim Korrespondenzautor angefordert werden.
